# Supplementary material for: Compositional variability of Mg/Ca, Sr/Ca, and Na/Ca in the deep-sea bivalve Acesta excavata (Fabricius, 1779)
Source: PLoS One. 2021 Apr 30;16(4):e0245605. doi: 10.1371/journal.pone.0245605 (PMC8087087; doi:10.1371/journal.pone.0245605)
Supplement: S5 Appendix — (PDF) [file pone.0245605.s005.pdf]

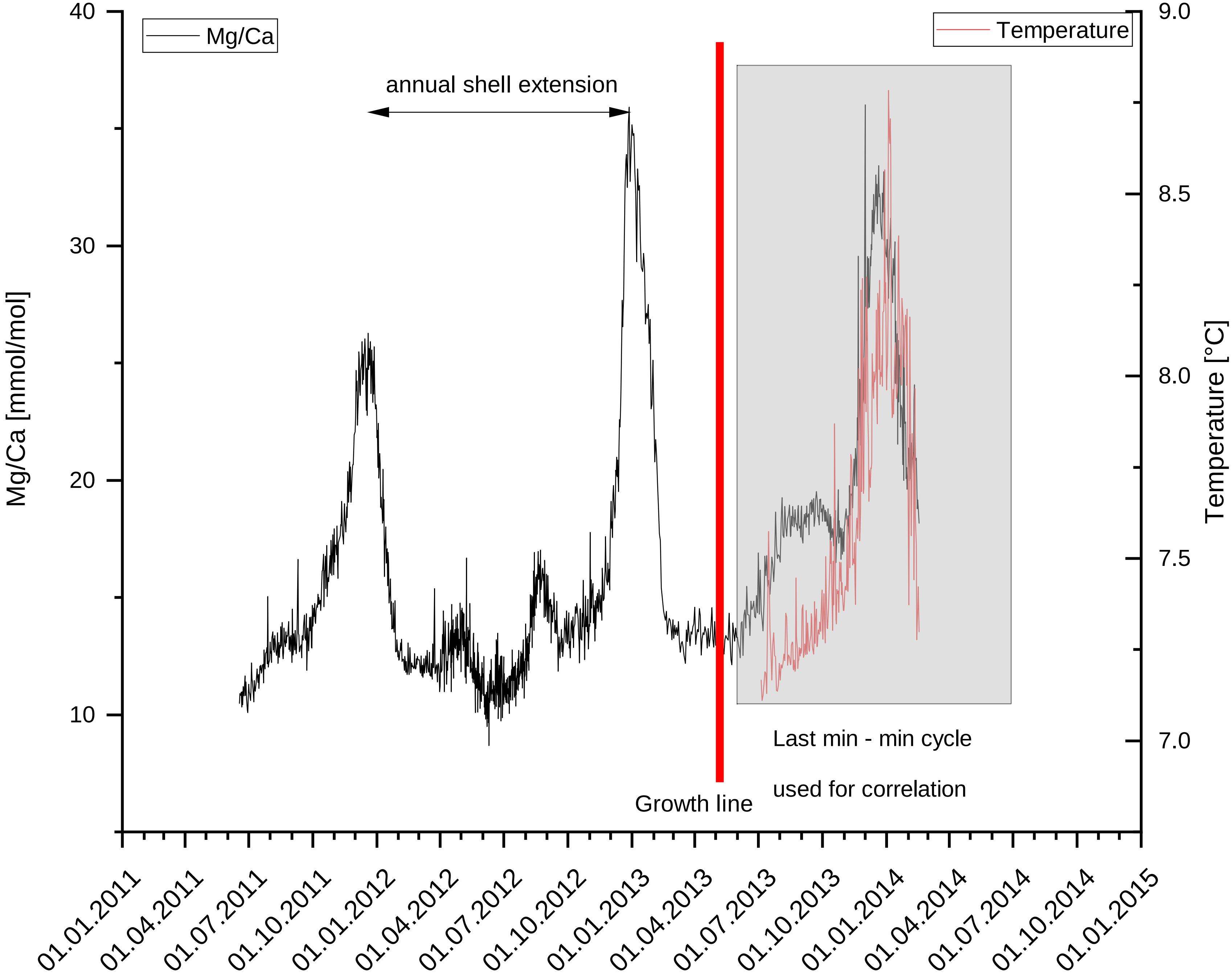

Process of Mg/Ca to temperature correlation. The last min - min cycle is considered to represent a yearly cycle.

Shell extension rates are determined by measuring the spatial distance between two Mg/Ca max. peaks.
