# Supplementary figures and images for: Compositional variability of Mg/Ca, Sr/Ca, and Na/Ca in the deep-sea bivalve Acesta excavata (Fabricius, 1779)
Source: PLoS One. 2021 Apr 30;16(4):e0245605. doi: 10.1371/journal.pone.0245605 (PMC8087087; doi:10.1371/journal.pone.0245605)

12R

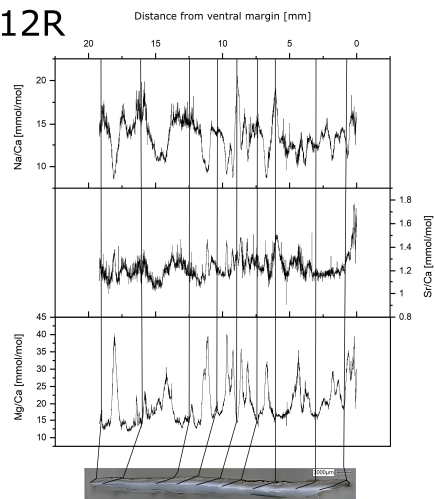

25R

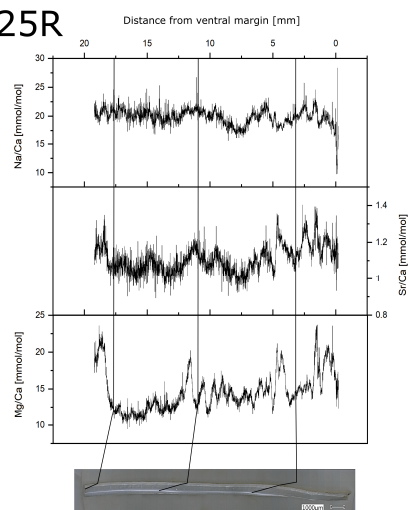

1R

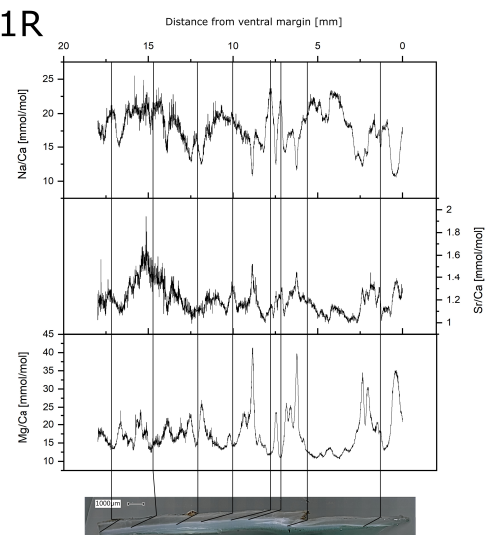

6R

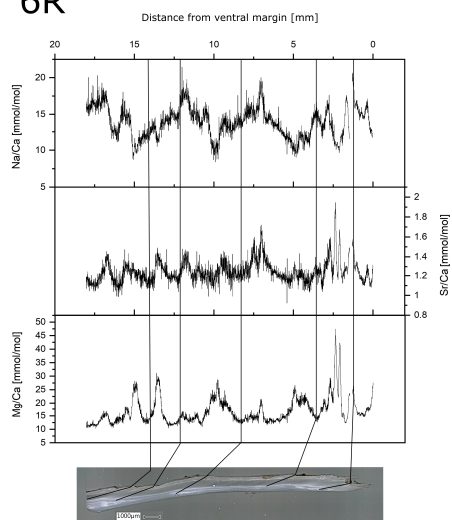

17R

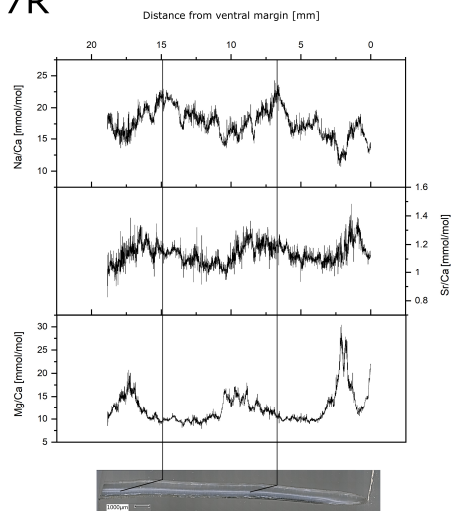

11R

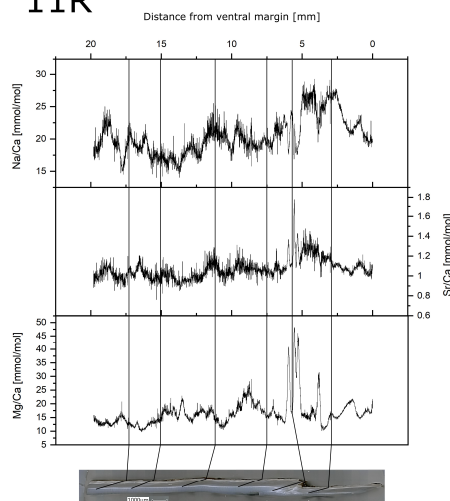

16R

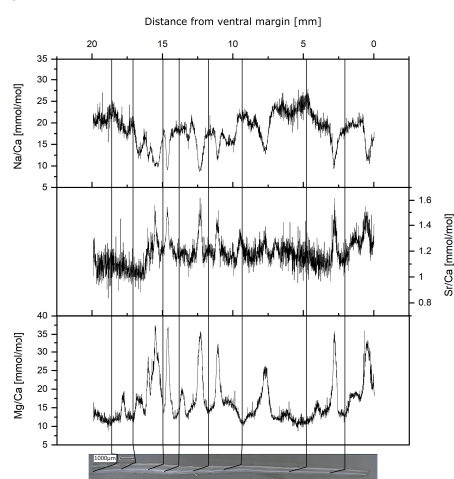

Supplement: S7 Appendix — (PDF) [file pone.0245605.s007.pdf]

# Samples from Sula reef

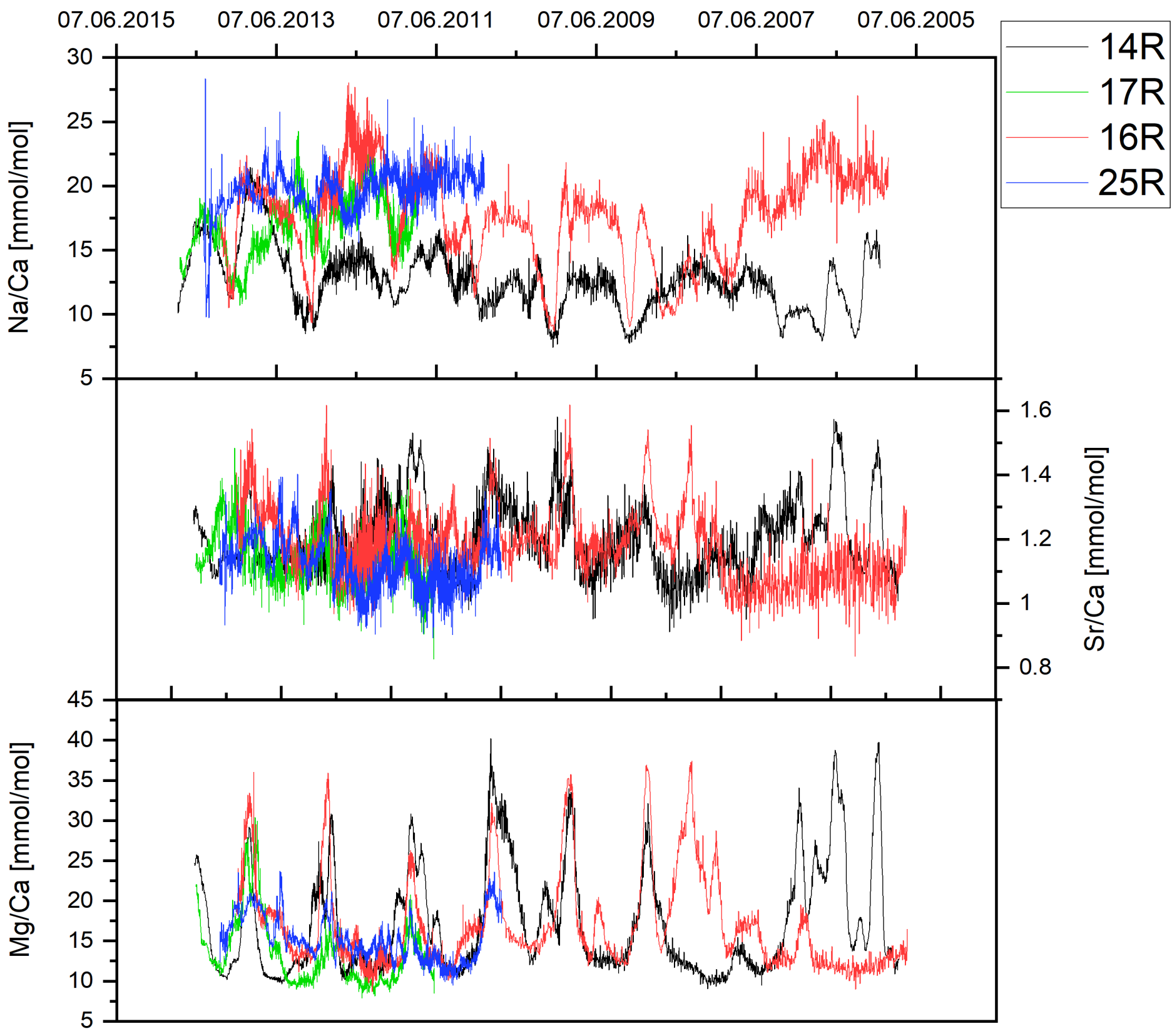

# Samples from Leksa reef

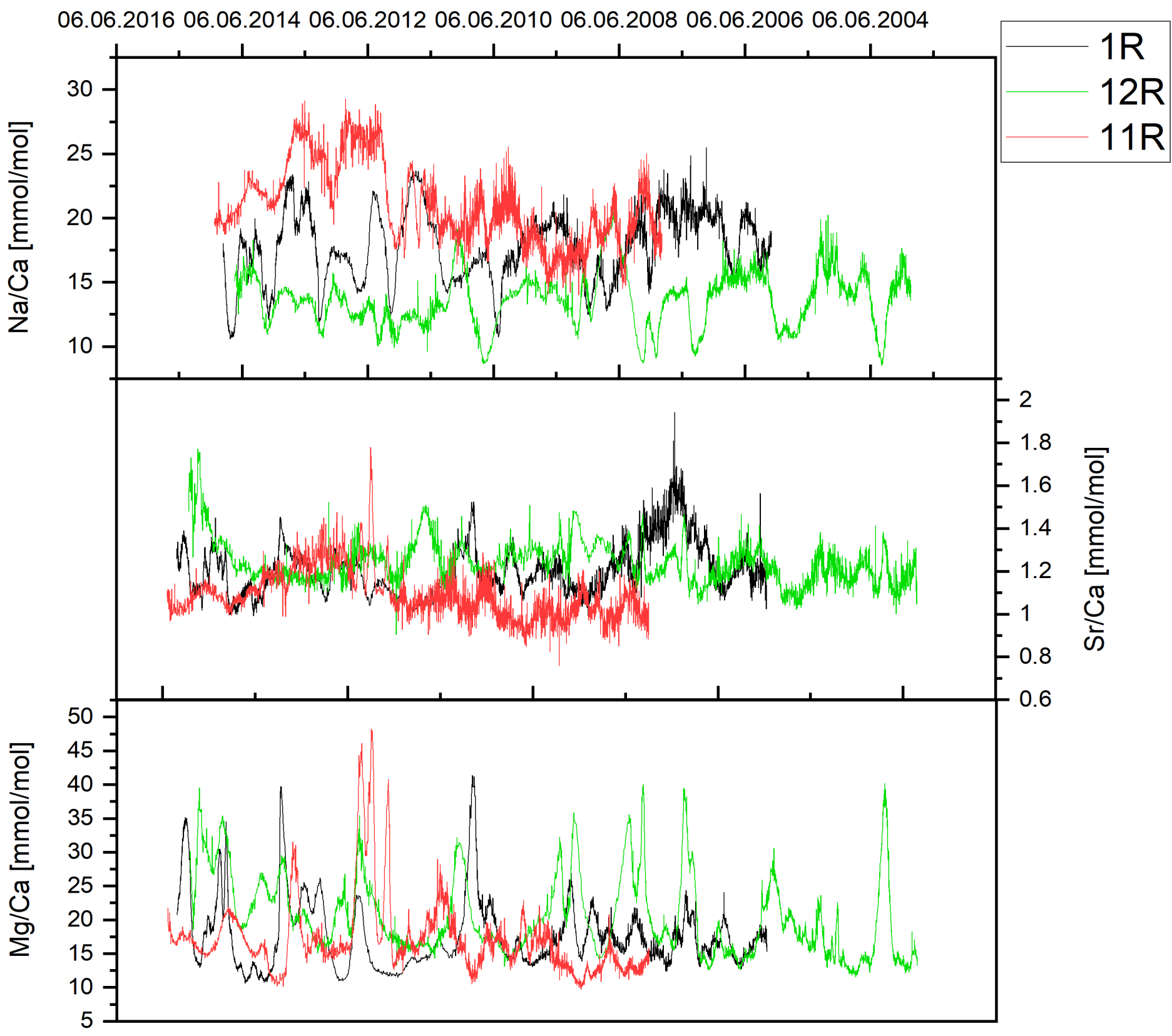

Supplement: S8 Appendix — (PDF) [file pone.0245605.s008.pdf]
