# Supplementary material for: Compositional variability of Mg/Ca, Sr/Ca, and Na/Ca in the deep-sea bivalve Acesta excavata (Fabricius, 1779)
Source: PLoS One. 2021 Apr 30;16(4):e0245605. doi: 10.1371/journal.pone.0245605 (PMC8087087; doi:10.1371/journal.pone.0245605)
Supplement: Attachment — Submitted filename: Reply to the reviewers.DOCX [file pone.0245605.s009.DOCX]

Reply to Reviewer 1

We thank Reviewer 1 for providing very constructive comments, which considerable

improved our manuscript. We are confident that we have revised our manuscript such that all

the reviewer’s questions have been addressed.

L36: Include information about the time frame of this with respect to carbon. Talk about temperature changes in a separate sentence.

L35: We included information about reservoir size and carbon flux.

“As the deep ocean is the largest reservoir for carbon with a fast exchange time (38000*10^15^ g C; 90*10^15^ g C/a) with the atmosphere, temperature changes in these water masses can have major influences on the global climate(1)”

L56: You need to add in more information about what IS known about seasonality in CWCs and why archives with well-defined growth histories are lacking in your study area and elsewhere.

L56: We added information why CWC are not suitable for paleoenvironmental reconstructions on a seasonal basis. Information about seasonality in CWC reefs can be found in L49- 54

CWC can be used for paleoenvironmental reconstructions as shown by several researchers (2–4). However, for seasonal reconstructions they are a poor choice due to their poorly understood growth increments (5)

L60: You need to provide the reader more information about how the growth patterns are simple and why that makes it a good potential archive of seasonal variations in seawater physiochemistry.

L60: Thank you for that remark. We described the process of shell formation in greater detail and pointed out how that helps to understand seasonal fluctuations.

“Bivalves usually produce consecutive growth increments that are visibly marked by growth lines. Major growth lines are formed annually due to growth cessation that occur at low temperatures or other environmental and biological triggers (e.g. change in food fluxes, rhythmic spawning events), which impede the bivalve from further calcification (6–8). First observation make a seasonal accumulation of growth increments in A. excavata likely (6). Different stages of growth increment completion in A. excavata that were collected at different times throughout the year can be observed, as well as cyclic repetitions of density changes in the shell (6), which are possibly controlled by temperature or salinity changes (9).”

L109: Be sure to include information about the analytical precision of all measured water chemistry parameters.

L126: We added the analytical precision of the temperature and conductivity sensors. Errors for turbidity, oxygen and fluorescence sensor are not given in the datasheets of the manufacturers.

L115: Write out the dates

L130: Corrected

L122 This sentence is confusing.

L136: Revised

“Utilized data consists of composite temperature measurements from 5 – 9°E/63.5 – 64.5°N, as such temperatures from locations to the northeast of the CWC study sites are included. Therefore, ARGO temperatures show lower minimum temperatures than recorded by the deployed landers as the ARGO data also includes lower temperatures from more northern sites.”

L142: Formatting issue?

L158: Corrected

L152: no need to capitalize here

L168: Corrected

L216-223: This content belongs in the discussion.

L340: Section moved to discussion

L286: Are these statistically significant?

L74: Yes, these are statistically significant on a significance level of 5%. We added a table with p-values as well as slopes for each elemental ratio.

L306: Move to discussion.

L378: Moved to discussion

L316: There needs to be more information about what is already known for this species regarding their growth increments, unless you're the first to work with this species? In general, you should add more citations to justify you hypotheses based on the results of previous studies.

L308: We are not the first to work with this species but the first the provide geochemical shell data. Until now there are only few studies available about *Acesta excavata* of which most are treating the biology of this species. We added as much information from other studies about growth increments in *Acesta excavata* as possible

L321: Have others looked at fluorescence in shells? What did they find? Need to add more information.

L312: We added results from another study. Mahé et al. 2010 found the same visibility of growth lines under fluorescent light in C*erastoderma edule* (10). Excitation wavelength was 460 – 490 nm which is in the same range of what we used (420 – 490 nm)

L327 *A. excavata* from this study area live at lower temperatures? Is it possible that they still shut down during winter at this specific location? Again, need more relevant citations.

L324 Upon further evaluation of the data we have to agree on your correction. The evidence we were stating is not strong enough to support our interpretation especially when we consider temperature/salinity as the trigger for microgrowth lines, as these show similar periodicities as the temperature/salinity variability.

Ultimately, we cannot give an exact answer on the effect causing the formation of growth lines as the thermal tolerance of *A. excavata* has not been researched. Spawning activity is discussed as a trigger for annual growth lines (9) however, it appears that this is just a covariance with changes in temperature (11,12) that trigger the gametogenesis/spawning activity (13)

L329: from where? Is this timing consistent everywhere?

See answer in L324

L332: I disagree. There are many factors that affect growth increment formation that require further discussion. You also need to better justify why you think they're not shutting down in winter, because I am not convinced.

See answer in L324

L352: Cool! Discuss in more detail.

L331: We added information about other species that show tide-controlled growth increments as well as possible controls through temperature and salinity.

L372: I would move this paragraph underneath the 'Further influencing mechanisms' section as the opening paragraph.

L372: We removed this section, as it does not fit the manuscript anymore.

L390 & L398: How are they affected? Why would organic matter affect the temperature reconstructions? Citations.

L374: We added more information about how organic matter could affect temperature reconstructions. In short: Organic material in shell material usually show greatly different elemental ratios compared to the carbonate. Magnesium is usually enriched in organic matter (14) which would lead to overestimated temperatures using the Mg/Ca temperature proxy.

L418: Why should changes in Na/Ca reflect changes in calcification rate?

Also, need to clarify if all this is true for all bivalves or for specific species.

L483: We deleted the whole section about calcification rate.

Changes in the calcification rate should be visible in Na/Ca ratios as sodium substitutes for calcium which induces a charge imbalance. These imbalances are compensated for by lattice defects. As these lattice defects are a function of calcification rate -> the higher the calcification rate -> more lattice defects -> more locations for sodium incorporation. As this is a mineralogical effect, it should be visible in every organism except it is supressed by other biological factors (15,16).

L427: The way this section is organized seems confusing. Reorganize to streamline and increase readability.

L366 – 490 We revised the whole section and inserted subsections to increase readability. We divided the section, based on possible influencing factors into four subsections. Furthermore, we shortened the section

L439: Why? Could this be due to shutdowns due to winter growth? Turbidity? Nutrient fluxes?

L407: We deleted this sentence. Increased current velocities could trigger growth line formation due to decreasing nutrient availability. Comparative measurements on growth rates from bivalves living inside and outside of seagrass meadows have shown that bivalves living inside these meadows grow faster. This was attributed to the baffling of the seagrass which increases the food concentration (17)

L449: Has anyone done a tank experiment for this species to test its thermal tolerance?

L434: We are not aware of any tank experiments to test the thermal tolerance.

L464: Throughout the manuscript you switch between spelling it out and abbreviating. Choose one and be consistent.

L464: We changed everything to abbreviations after the first mention

L478: This section is repetitive. Either remove redundant information or move all discussion of Mg/Sr ratios for temperature reconstructions to this section (my preference).

L491: We deleted this section but added another short section about how the combination of two elemental ratios can be used to overcome vital effects.

L506: Stress seems like a really vague way to describe changes in temperature, turbidity, nutrient source, pH, spawning, which can all cause stress. Because each of these factors have a unique effect on bivalve growth and metabolism, they should all be treated as separate and discussed individually and in more detail than they currently are.

L399: Thank you for that objection. We added a section about how stress and the bivalves response to it can potentially changes the elemental signature of the shell, independently from what might cause the stress.

In general, bivalves respond to a stress situation by closing their shells to reduce the connection with the stress trigger. This causes metabolic products such as ammonia or malate to be concentrated (18). Malate was shown to increase the amount of Na and Mg incorporated into the precipitated calcite when present in the calcification fluid (19)

The concentration of ammonia and CO_2_ in the organisms does also change the pH of the body fluids (18,20). This can both directly affect elemental ratio signature in the precipitated shells (21) as well as indirectly through selective dissolution of Ca from the already precipitated shell (22).

Additionally, the heart rate of bivalves changes during shell closure and ATP concentrations decrease (20,23), possibly indicating changes in the metabolic activity of the organism. The decrease of ATP can have effects on active ion transports such as the Na/Ca-ATPase which would lead to a decreasing influx of Ca-ions into the calcifying space.

L517: Who has studied them? Cite!

L472: We added proper citation

L560: But above you say these cannot be the only factors. What else controls Na/Ca?

L471: We revised the whole section. Temperature and salinity on their own cannot account for the observable Na/Ca variability. But combined, these parameters can account for up to 66% of the observed variability. Additional, controls are probably provided by the concentration of organic material, where sodium is enriched (24,25) and changes in the calcification rate (15).

L1020: Make all elemental ratios panels the same height and make the bottom shell images bigger

We adjusted the image after the reviewer’s recommendation

L1020: Red trend lines are difficult to see. Consider changing the symbols to lighter colors and making the trend lines in bolded black.

We changed the black datapoints to grey and the trendlines to black.

Reply to reviewer 2

We want to thank Reviewer 2 for their time to review our manuscript and thank him for the comments he provided. We revised major parts of the manuscript to address the remarks of the reviewer. We included all the Mg/Ca data into the temperature correlation instead of opting for fix points that might be entirely subjective. This resulted in a weak but still significant correlation of Mg/Ca with temperature. However, as the reviewers objected, the large scatter makes Mg/Ca ratios unusable as a proxy for temperature reconstructions in *A. excavata*.

Multiple linear correlation with temperature and salinity as predictor variables, however can predict the elemental variability to a large extent (Up to 79% for Mg/Ca, 66% for Na/Ca and 59% for Sr/Ca.

We describe further control mechanisms, such as changes of the concentration of organic material or biological responses to stress which could provide further controls on the incorporation of the investigated elements.

We also show that combining different elemental ratios such as Mg/Ca and Sr/Ca can be used to account for some of the vital effects. Even though high scatter still prevents us from using Mg/Sr ratios for paleoreconstructions.

We hope that the revised manuscript satisfies the objections mentioned by the reviewer.
