# Supplementary material for: Compositional variability of Mg/Ca, Sr/Ca, and Na/Ca in the deep-sea bivalve Acesta excavata (Fabricius, 1779)
Source: PLoS One. 2021 Apr 30;16(4):e0245605. doi: 10.1371/journal.pone.0245605 (PMC8087087; doi:10.1371/journal.pone.0245605)
Supplement: Attachment — Submitted filename: Response to reviewers 2.docx [file pone.0245605.s010.docx]

**General comments**

We are thankful for the comprehensive reviews we received. They were very helpful and helped us tremendously in improving our manuscript. We reworked large parts of the manuscript and incorporated the reviewers remarks as best as we could.

We modified the manuscript to meet the journals requirements, added a section about permits for sampling and added funding information as requested by the editor.

The reviewers comments are given in black with our response in red.

**Reply to Reviewer 1**

I am uncertain that the small number of specimens is enough to draw any conclusions here.
Also the small variability in temperature seems far too minute to get any good patterning especially if the vital effects are not clearly understood.
But I abstain from any judgement as I am not familiar with deep sea bivalve records.

We thank Reviewer 1 for his review. With 4 specimens per location, we have a similar amount of specimens as other comparable studies ((Lazareth et al., 2013; Marali et al., 2017a; Schöne et al., 2011)

**Reply to Reviewer 2**

We thank the reviewer for these very helpful comments which considerably improved our manuscript.

“This is an interesting study with some intriguing results that the paleoceanography and sclerochronology communities would find useful. That said, the paper lacks structure and direction and the reader does ……..”

“The beginning of the introduction does not appear to be relevant based on the abstract, some very wide and irrelevant statements are made. It gets better after line 37 and reference 5. For the introduction, do not just make broad statements that are generally true but may have no relationship to your conclusions and your study………”

We deleted lines 30 – 41 of the introduction as well as revising large parts of it by adding more general information about the investigated species. We also added more information about the purpose of this study and what we hope to achieve.

Furthermore, we also added a section about the compositional differences between the two shell layers (L354-374) as well as about the oxidative cleaning procedure (L375-387) so that the meaning is not lost in the manuscript.

We also specified that we recorded and used *in-situ* environmental data (L83-85)
This study has in situ temperature and salinity. This is key part of the study and makes this study unique. However, there are some key details left out. How often were the measurements taken? hourly, daily? The authors also mention ARGO data was used, but this data and the in situ data are not shown in the paper. Furthermore, the authors never discuss how time in the shells was assigned for comparison to the in situ instrumental data. This is important, and will change all the correlation results, among shells and a with shells and instrumental data.

We added information about measurement frequency (L139-142), the depth of the landers (L137-140) and the sampled specimens (L96-99). We also prepared a new figure, that displays both the lander data and the ARGO data (Fig. 3). Time assignment was described in the original manuscript in L271-282 (now L276- 288).

Methods: How was soft body removed from the dry shells? Sonication, ultrasonic cell disruptors? Were the slabs in epoxy cut more? If so how? What precautions where taken to ensure cleaning and polishing were not altering shell chemistry? Can you give the water depth the shells are from and depth of the landers recording ocean conditions?

We added additional information about the cleaning procedure (L103-105). After embedding in epoxy the samples where not cut any further only ground and polished. Specific precautions were not taken. We are not aware of any studies that suggest chemical alteration induced by polishing or grinding. We used silicone carbide and diamond paste for grinding and polishing which should not interfere with the measurements. Considering the cleaning of the shells, we kept the time in which the samples were submerged to a minimum. Information about lander depth can be found in L137-140
The standards for LA-ICP-MS accuracy and precision: The accuracy is a large number, was the data adjust for the accuracy offsets? What Ca isotope was used for Elemental/Ca? Was the same Ca isotope also used for the internal standard? How was the ICP-MS data processed? Many labs use WaveMetrics IgorPro with Iolite software. The study only samples a small part of the shell, can the whole shell be sampled and how old does this species get?

The LA-ICP-MS data was processed with a custom Excel sheet. The data was not corrected for the accuracy offset. The offset from reference value and RSD (Mg/Ca=5% (RSD = 3.7%), Na/Ca=6% (RSD = 5%), Sr/Ca=2% (RSD=4%) is within the same range as in other comparable studies (Marali et al., 2017b (Mg/Ca = 6%, Sr/Ca = 2%, Na/Ca = 8%)(Schöne et al., 2010 (Mg/Ca RSD = 7.2%, Sr/Ca RSD = 7.8)) where no correction is applied. ^43^Ca was used as both the internal standard and for normalization (L157 in the original manuscript). We also have whole shell measurements from some of the specimen but did not include them into this dataset as they do not provide much additional information within the framework of the study. Typical lifespan ranges from 50 to 80 years (López Correa et al., 2005)

Detailed items: Note some of these items (spelling, grammar, useage, etc.) are reoccurring problems in this paper. The authors should use find and replace to fix throughout the manuscript and carefully edit and revise.
Line 11 revise to “…cold-water coral reefs…”

Corrected
Line 13 revise to “…instrumental seawater parameters such as…”

Corrected
Line 15-17. This is redundant, if correlation is weak, the explained variability will be low. R^2 = variability explained. Revise to be more concise and give the r value, with the n and p-values. Do this for all reported correlations throughout the paper (such as lines 18, 19). Same for sensitivity, put this value in parenthesis with its uncertainty, so the reader knows what you are referring to.

We revised the section by adding the correlation values directly to the text or referred to an according table.

Line 20 Do you mean the correlation of elemental ratios with extension rate was the highest? The “control” is assumed, correlation is not causation. Revise.

Revised to correlation (L24)

Line 21 The term “vital effects” is used by many authors and the definition is not clear, it could mean biological effects, reproduction effects, species effect, depth effects, calcification rates, etc. Be more specific, just say “growth-related effect”, determine by linear extension. Line 23-25 shows that these “vital effects” are quite varied.

We changed the wording to growth related effects. We note that vital effects do not include depth effects, as vital effects describe internal biological fractionation processes (Pérez-Huerta and Andrus, 2010)

Line 22 Clarify “Multiple linear regressions” did you do regression many times or once? If once revise to “Multiple linear regression”.

We only did the regression once per sample.
Line 22 “a large amount of elemental variability” Be specific and give and exact number.
Additionally throughout the paper, avoid using descriptive adjectives (i.e. large, low, etc.) for numerical analysis. These adjectives have no meaning, what value do you assign to “large” or “low” better to give the value and let the reader decide if the value deserve the descriptor. This removes any bias the authors place on their data.

Thank you for that comment. We deleted descriptive adjectives wherever possible and added values or references to tables.

Line 23 Calcification rate does not equal linear extension rate. Calcification rate = linear extension rate x density. How do the authors account for density differences that would impact calcification rates?

We completely agree that calcification rate, growth rate, linear extension rate, etc. are often used interchangeably when they are actually not, which we also specified in the text (L516). We did not account for density differences as we do not report calcification rates and only speak of linear extension rates. We only discuss calcification rates in the context of other studies.

Line 26-28 This needs to be explained better. Just using Mg/Sr does not mean you get around growth-related effects. Revise “combining different proxies such as Mg/Sr ratios” Proxies are some property of an archive that can be a “proxy” for some environmental parameter of interest. You mean “combining different elemental concentrations such as the ratio of Mg to Sr…” Stating Mg/Sr ratio is like saying ratio twice since it is given as a ratio with /.

We deleted the sections about Mg/Sr as there is not enough evidence to support that conclusion.
Line 30 Revise “Intermediate water masses have been shown to respond with highly sensitivity to climate forcing”

Deleted.
Line 32 use a comma before “which”, always. Fix throughout. Additionally, double check the “that” and “which” are used properly each time.

Fixed.
Line 31-33. This sentence confuses me. Be more specific. What do you mean by “atmospheric climate changes” and “small scale effects” ? CO2? Water vapor, wind? Waves?If your archive-proxy is intermediate water why not just say they reflect intermediate water oceanic conditions. From your abstract, the proxies are argued to be co-varying with water temperature and salinity. What does these two properties have to do with the atmosphere?

We deleted this section upon your recommendation.
Line 47-48 This sentence is confusing, revise “Water flow velocity and indirectly also its effect on food supply to the corals provide further controls.” Something is missing after “indirectly”. What does “its” refer to? Perhaps the “also” is not needed, makes more sense without also? What is being controlled? In general, avoid using “it” and its forms, be precise and tell the reader what you are referring to. Remove “it” throughout by revising the sentences.

“Its” referred to water flow velocity. We changed the sentence to increase readability and removed the use of it throughout the manuscript.

Line 52-53 Shallow water reef corals spawn with Moon phases. I would not be surprised if cold water corals do the same, see link.springer.com/article/10.1007/BF00301193.

Of course, there is a lot of potential triggers for coral spawning including lunar phases, salinity, temperature, food supply, solar insolation among others (Brooke and Järnegren, 2013 and references therein). Ultimately, the exact triggers are not known. In the study the reviewer referenced, the control through lunar cycles is explained to happen through the light intensity of the moon. This effect can be certainly excluded for corals that inhibit water outside the photic zone. In deep water corals, a lunar control can only be exerted through different tide cycles. The authors also state that temperature does not control the spawning activity of the observed corals because absolute temperatures are different by 5°C. It is still possible that not the absolute temperature triggers the spawning activity but the beginning decrease in temperature as the spawning period in two of the observed reefs happen right after the temperature maximum.
Line 56 The reference refers to just one cold water coral species Lophelia pertusa, and there are jundredss of these corals, so how can you extrapolate from one species to all of them? Less work has been done with cold water corals but does not mean they all do not make good archives of past conditions. This sentence is also missing a period at the end. Most of your references are for L.pertusa but there are other cold water corals that can be used for reconstructions such as bamboo corals (see doi:10.1016/j.gca.2018.12.027, doi:10.1016/j.epsl.2019.11575, doi:10.1029/2011PA002260, doi:10.1029/2010GC003443, www.sciencedirect.com/science/article/pii/S0016703711002808, Desmophyllum www.sciencedirect.com/science/article/pii/S0012821X12003652, Adkins 1998 www.sciencemag.org/cgi/content/abstract/280/5364/725, dx.doi.org/10.1016/j.gca.2011.02.019, www.sciencedirect.com/science/article/B6V66-50PVG4J-2/2/4a35c81fe923000631c5a6f4b211ef50 and others.

Thank you for that remark. We have specifically targeted *Desmophyllum pertusum* for comparison, as this is the main reefbuilding coral in our sampling location (and in general). We added some of your references to show that other coral species can certainly be used for reconstructions.
Line 57-58 Reference #25 is not a coral paper but an interlab comparison paper and not an appropriate reference.

We assume that you mean reference #22. We deleted this reference.

Line 58-59 I do not argue that bivalves are not valuable archives for reconstructions. Yet deep sea corals are still a work in progress and it is not time to give up on them. I would also argue they have simple growth patterns like a tree-rings. Additionally, bivalves tend to grow slower in colder water making seasonal reconstructions more difficult except for the intervals in the youngest parts of the shell where growth is the fastest or using laser ablation ICP methods, which can also be used with deep sea corals. As the shell ages, the seasonal resolution becomes more difficult to obtain and many researchers use annual resolution to produce long continuous reconstructions or extension rate themselves for the reconstruction. Be careful of negatives you describe for any archive-proxy because they can also impact your study!

It was not in our interest to claim that deep sea corals are bad archives or inferior to bivalves. Just as you say research is still very much in progress and is just at the beginning. We adjusted the wording and added information about other corals that show more promising results (L54-56)

Line 61-65 What about bivalves from the tropics and subtropics? They still have growth lines and they can be annual. Give a reference that states growth lines are annual. Additionally, if you just refer to the species in your study, that would be better than generalizing for the tens of thousands of bivalves species that exist. Do all bivalves have visible growth lines and all they all annual? I bet I could find some that are not. Revise to be more specific and do not make class wide generalizations.

We reworded the section to be more specific. L56-L58 “. A first study revealed cyclic repetitions of density changes in the shell and regular growth increment spacing, indicating a rhythmic, possibly annual control of shell deposition (López Correa et al., 2005)”
Line 65-66 This is an incomplete and confusing sentence “First observation make a seasonal accumulation of growth increments in A. excavata likely.”

Lines 66-69 Another sentence needing revision for clarity, suggestion “Different stages of growth increment development in A. excavata, which were collected at different times throughout the year, have been observed, as well as cyclic density changes in the shell, which are possibly controlled by temperature or salinity changes.

We added a reference, deleted the generalizing statements (L54-L64) and added some more specific information about this particular species (L57-L69)
Line 70-71 Reference needed. doi.org/10.1016/j.chemgeo.2019.01.008 suggested otherwise.

This sentence should only act as a general information. More bivalve specific information is incorporated in subsequent sentences.
Line 78-81, For Na/Ca also see www.sciencedirect.com/science/article/B6V6R-4894W8C-89/2/9bfe9e583e5fa7fc01c95e81a6604064, and doi:10.2343/geochemj.1.0067.

We added your suggested references
Line 86 delete “very”. This word should be avoided in technical writing unless it adds to the sentence meaning. Very is an overused word. Whenever you’re tempted to use it, try dropping it to see if any meaning is lost.

We deleted “very” here and in multiple other locations.
Line 105 Do you mean the slab surface was polished? How were the surfaces polished?

Yes, the slab surface was ground up to 9 µm grit with silicone carbide pads and polished up to 3 µm grit with diamond lapping paste
Line 109 and figure 1. Use of “commissure”, do you mean where the two valves meet or the shell edge? This term is usually used with brachiopods. Just say ventral margin or edge of the shell.

We are referring to the ventral margin and changed it accordingly. Commissure is also used in context with bivalves (Bailey, 2009; Vermeij, 2013). However, we agree that the use of commissure in this particular context is wrong.
Line 100 What is “the ear of the bivalve”? Bivalves do not have ears. See Bailey, 2009: Journal of Paleontology 83, 493-495.

We are sorry for this false translation. In german, the bivalves auricle is usually referred to as “ear”.
Line 112 “location marked in A with a dotted square” The top image is marked with an A, but there is no dotted square in in the figure. There is a dotted rectangle inside the solid rectangle, is that what you mean? The image in B looks like the orientation is from the Umbo to the ventral edge not along the growth lines. Use a A-A’ to mark the orientation and location of B.

We added coloured lines to make the figure better understandable.
Line 113 space between number and unit, check throughout. Line 162 space between word and reference, check throughout.

Corrected
Line 114 Revise “the opposing side”, you mean just the other side, not both sides, correct? Replace “surfaces” with “slab” since that is the term you used earlier or “slab surface”. Does this mean you cut two slabs from each shell or did you just cut them in half? I assume you have to cut thinner slabs to fit in the laser chamber.

We cut two strips for each specimen to get matching surfaces.
Line 114 Define LA-ICP-MS at first use, it is defined in line 147. Same for all abbreviations, check throughout the paper. Line 221.

Corrected
Line 180 Spelling mistake “recorded”. Cite the figure you are referring to.

Corrected
Line 181 Summer and winter have more than one month, use “months”.

Corrected
Line 180-181 Revise “Temperature varies from 7.7°C in the summer months to 8.5°C in the winter months.” The summer is colder than the winter? Odd. Is the Sula reef the same? OK, I see the explanation in lines 184-186, move this up to the temperature sentences.

Corrected
Line 182 I do not think you mean “The Sula reef shows a temperature variability of 7°C – 8.8°C” meaning the temperature can vary by 7 to 8.8 degrees in a year but the temperature changes from 7ºC to 8.8 ºC during the year. Where are these results and data in the paper? There should be a figure and a data table for these data. If the data is already publicly available, then a citation is needed with the internet access address.

We added figures for this data and a data table to the supplements (S6). We changed the wording for the temperature variation
Line 183 Be consistent with to or –. “33.5 to 36 g/kg in the Sula reef and 34 – 36 g/kg in...”

Corrected
Line 191 revise “The different shell layers are not easily identifiable…” layer is more descriptive of what you are describing.

Corrected
Line 220-223 Means are not robust against outliers. What do are the median values?

We added median values. While the median values are lower the difference between the two reefs is equal to the mean values.

Isn’t Fig 2 the data from the shells, not fig 3 which are scatter plots. Is the mean in line 221 for all shells or just one, clarify.

The mean reported in line 221 is for all observed shells.
Figure 2 I do not see the yellow line for the laser path. Perhaps use another color like blue?

We fixed that error
Figure 3 This is a scatterplot of the elemental data for just two shells. Where is the data for the eight shells, like Figure 2? This needs to be included somewhere in the paper. There are eight shells measured, where are those plots? Establishing the annual cycle in the archive is important, as you point out in our introduction. So where is the proof of annual layers for these shells? At least have a table with the descriptive statistics for each shell, then you do not have the write it out in the results. I cannot see any “hollow” marks mentioned in the caption. How were outliers identified i.e., what statistical test did you use? How were the “fit functions” determined? With this many data points, any correlation will be significant, but not if you assess correlation with degrees of freedom. See Rodgers 1988 www.jstor.org/stable/2685263 and EBISUZAKI 1997 doi:10.1175/1520-0442(1997)010<2147:amtets>2.0.co;2.

The data for all the measurements is in the supplements. We also added the according figures to the supplements (S7). In response to other reviewers comments we deleted the scatterplots and any connected text passages as they do not add valuable information. Like suggested we added a table with descriptive statistics
Line 226 “A correlation between Mg/Ca ratios and Na/Ca ratios is visible” where is this visible? Visible is not very scientific, give a correlation value with n and p-values. This sentences jumps to Na/Ca which has not been described yet. Talk about correlation among El/Ca after presenting results for each El/Ca.

See former answer
Line 229 is this for all shells? See previous comments for Mg/Ca

This is for all shells combined.
Line 230 How was no difference determined? What statistical test did you use?

We used a regular t-test. We are aware that the t-test needs normally distributed data, which our data is not. However, the central limit theorem states for a large enough n (>100) X approximates a normal distribution (Bergström, 1949). We also tested the data with a Mann-Whitney U-test, which gave the same results.
Line 231-233 What statistical test were used to evaluate correlation? Add a correlation table for the shells and El/Ca. For an example of how this can be done see Stephens 2004 doi:10.1029/2004GL020343.

As said earlier we deleted these sections from the manuscript.
Line 238-239 This is an interesting result, I like that you tested this. How many shells did you test this for? But where is the data and figure? Whenever you use the term “significant” there needs to be a statistical test to determine the significance. How was significance tested? Provide those details.

Unfortunately, we only tested this for two shells. We added a data table for these results. Significance was again tested with a t-test
Line 243 Microgranular shell section Move this section up to the beginning of the results since you already started describing it. How are the growth rates different among these shells? Could growth rates be driving the differences you noted in this section of the manuscript?

We added a table with the determined linear extension rates. Growth rate differences might play a role in the described differences but we cannot comment on that for a lack of knowledge how extension rates differ among the two shell sections. Possibly, the different crystal morphology is the driving force behind the observable differences.
Line 252 Correlating element/Ca data to instrumental data
Before jumping to correlation, where are the growth rates of the shells described? I would put all the physical properties of the shells first in the results section. Then geochemical results, cleaning results, replication results – intra and inter reef. Then you can move on to correlation between the geochemistry and environmental data.

We revised the sections based on your suggestions.
One of the largest issue of correlating shell geochemistry to environmental data is time assignment in the archive, this is also a large source of error. Some researchers with correlate or “calibrate” the geochemistry with environmental data using just the max and min of a seasonal cycle -this is the easiest to do. Coral paleoclimatologist use Analyseries or QAnalyseries (sites.google.com/site/geokotov/software) for time assignment of many years to an SST record. Some sclerochronologists use von Bertalanffy growth curve to help with time assignments. Problem with LA-ICP-MS data is you have a lot of data that makes time assignment cumbersome but doable. Please give details on how you are assigning time to your shell data. If you assume Mg/Ca is correlating with temperature and you sue that for your time assignment, it will have the highest correlation! Please state the basis for make this assumption and test doing time assignment with the other geochemistry to see if the results vary (they should). By using Mg/Ca for time assignment, you have bias the correlation analysis. If you can use the growth lines to assign time then you can remove or reduce this bias.

We completely agree that the time assignment procedure is very important. In general we were using the min-max method you were mentioning with Qanalyseries. We assume that Mg/Ca is so some extent controlled by temperature, because the underlying mechanism is not controlled by an organisms biology but by thermodynamic effects (Mucci and Morse, 1990). However, we used growth increments for the majority of the time assignment. Only if the growth lines were cryptic, we utilized the Mg/Ca ratios as a further help. This method is also used in Halfar et al. 2000.

We are not sure why utilizing other geochemical ratios should lead to different results. If we assume that Sr/Ca is similarly controlled by temperature (Stoll et al., 2002), then utilizing Sr/Ca ratios should results in the same chronology (which it does).

Lines 257-265 Please add a correlation table to show the reader these results. The shells with low correlation, could that be a time assignment issue? Look at replication. Does the correlation improve if you build a master record with two to eight shells? The signal to noise ratio should improve as you average shells together and thus the correlations improve, if there is really a causal relationship.

A correlation table is in the manuscript (L305). Since we only have data for 14 months we cannot use a longer Master record. The shells with low correlation can be an effect of temporal misalignment but we don’t think that is the case. Otherwise the multiple linear regression should show an equally low correlation, which is not the case.
Line 271-275 now it is mentioned that data were tuned. Shouldn’t this be mentioned before the correlation analysis?

The temperature and salinity correlations was not conducted with the ARGO record but the lander data.

Line 276-282 Can you provide a citation for “nonlinearity of bivalve growth”. Does this happen within a year? Or do you mean on longer time scales? How did you “tune” the data? What software program did you use? Did you interpolate to get the geochemistry to a time scale? Include a plot of your raw shell data and your tuned shell data in your paper or appendix.

Since we tuned our data only using the peak Mg/Ca values, we assume a constant growth rate between two fix points (which is probably not the case). We used Qanalyseries for this task without interpolating any data points. A citation for the non-linearity of shell growth would be De Ridder et al., 2004.
Line 280-283 Distance in time-geochemistry or growth lines in the shell? How many years do you have per shell? Maybe 6 from fig 2? Is this enough years to assess a relationship between extension rate and geochemistry statistically? Have you extension data per shell in your paper as well.

The distance was measured from growth line to growth line. We added a table with the extension data to the manuscript. In general we have 4-8 years per sample.
Table 2 How was p values assessed? # of data points or degrees of freedom? With LA-ICP-MS data there are thousands of data points so any correlation will be “significant” but you should assess degrees of freedom and re-determine the p-values, see EBISUZAKI 1997.

We used degrees of freedom for significance tests
References need to be editing for spelling, proper capitalization for proper nouns, species names should be italics, [internet] removed and doi given instead. Access dates removed, etc.

We corrected the references according to your suggestions

**Reply to Reviewer 3**

We want to thank Reviewer 3 for his useful comments. We modified our manuscript with regards to the remarks by the reviewer.

“Abstract - - “Our results may still hold promise that A. excavata may serve as a paleoceanographic archive
for intermediate water masses despite the relatively strong vital effects, as these can potentially
be accounted for by combining different proxies such as Mg/Sr ratios.” Add a new sentence in the abstract, …more work is needed to confirm this idea.”

We deleted this section, as we think there is not enough supporting evidence.

Line 56- missing period at end of sentence.

Corrected

Line 58-59- some would argue that a compressing record with ontogeny is not simple.

We agree with your comment and deleted that statement.

Line 73- ref 32 is certainly out-dated. The study has never been replicated, either. If you are going to list one study that seemingly work for M. edulis, be sure to include other studies (e.g., Wanamaker et al., 2008) that show that the errors are very large using Mg/Ca thermometry and that vital effects seem to be dominant.

We included your suggested study into our reference list and modified this section to make it clearer that Mg/Ca and Sr/Ca based proxies in bivalves are heavily influenced by vital effects.

Line 78- see discussion by Wanamaker and Gillikin (4.3 The use of element to calcium ratios in bivalves as environmental proxies) on this very same issue. It would be good to cite this study here. And many of the references used there are more up to date. The literature review on bivalves in this section need to be a bit more up-to-date.

We added your suggested study to the references

Line 260. Capitalize Line.

Corrected

Line 399- Should be Wanamaker and Gillikin

Corrected

**Reply to Reviewer 4**

The comments and remarks provided by reviewer 4 helped us greatly in improving our manuscript. We included them to the best of our knowledge and hope that this modified version fulfills your remarks.

“First, I grateful the chance to read this paper. In this study, Mg/Ca, Sr/Ca and Na/Ca ratios were measured on live-collected bivalve Acesta Excavata (Fabricius, 1779). Even though, none (or very poor) correlation has been found….”

We have revised our manuscript and deleted the section about Mg/Sr as there is not enough evidence to support that conclusion. We also improved the way conclusions and implications are presented to make them better accessible.

“The manuscript is not very well written, and the author should be check grammatical mistakes or inconsistencies. For example: L. 56. Point after reconstruction (it is the end of the sentence). L. 260: Four with capital letter (it is the beginning of the sentence……”

The revised the language with help of a native speaker (Co-Author D. Evans).

Figures:
- I suggest to add a new figure including a map and a picture in order to show where the shells were collected.

We added a new figure with the sampling locations.
- Figure 2: You must include the series obtained from all shells, not only the results that you have obtained from one of them.
We added these figure as supplement S7
- Figure 3: I guess that they have included dataset from all shells. Is that correct? If not, results obtained from all shells should be added.

The figure included all sampled shells. We deleted the figure nonetheless as it did not add valuable information to the manuscript. It can be easily recreated from the included data.
- Figure 4: Improve this figure. The resolution is very low. Besides, add the scale.

We improved the image quality and made the scale better visible

Specific comments:
- L. 2: Please, add "(Fabricius, 1779)". Authors should include this information in the tittle, in the abstract, as well as the first time that they write this species in the text. They also must include the complete name of the species (Acesta excavata) the first time that they cited this taxon in the abstract and in the introduction.

We added the proper citation to the manuscript (Title, L13, L57)
- L. 13: Please, specify in the abstract what technique you used to measure this trace elements, i.e., LA-ICP-MS.

Corrected
- L. 62-66: Authors must include more information about growth patterns of this species if this is available. How many days throughout the year this species grows? What is the longevity of this species? Information about the effect of the ontogeny in the growth patterns is available? Indicate when the sexual cycle occurs? Gametogenesis? Spawning? Is there a relationship between sexual activity and growth stoppages?

We added more information about the bivalves biology (L57 – 69). Unfortunately, information about this species is scarce as this is one of the first studies to research the species.
- L. 90: Please, specify here what technique you used to measure these trace elements.

Corrected (L79). We used LA-ICP-MS on an Element XR MS with an ArF Excimer Laser system from Resonetics
- L. 105: Please, explain with more details what material and methods you used for polishing shell section. Glass plates? SiC grit powder?

We added a more thorough explanation about sample preparation. In short: We ground the samples up to 9um grid with silicone carbide sandpaper and followed with polishing up to 3um grid with diamond based lapping paste on a lapping towel.
- L. 146: How do you know this calcium carbonate layer is calcite and not aragonite? You must investigate the shell mineral composition, for example using X-ray diffraction (XRD) or, at least, referencing a previous study where calcium carbonate composition of this species would have previously been investigated.

We added a citation. Shell chemistry was tested by López Correa et al. 2005 with feigl solution and XRD measurments.

- L. 181: Why do you have warmer temperatures in winter? This is a little strange for me and maybe for other readers too. Please, try to explain this. I think that it is very normal and of course that there is a very simple explanation, but it is strange.

We can understand the confusion. An explanation was given in L184-186 in the orginial document (now L186). This is caused by the replacement of the cold and brakish Norwegian coastal current (NCC) by the North Atlantic current (NAC), which is warmer and saltier. During summer south-westerly winds induce a downwelling of the NCC, leading to cooler temperatures in the bottom water. During winter and spring, northerly wind and icnraesed meltwater input displace the NCC upwards, which allows the warmer NAC to replace the bottom water (Jacobson, 1983; Sætre and Ljøen, 1971; Sakshaug and Myklestad, 1973)

- L. 204: The same that previously. How do you know that layers are composed by aragonite or calcite? Please explain that.
We added a citation. Shell chemistry was tested by López Correa et al. 2005 with feigel solution and XRD measurements.

- L. 205-206: “These lines coincide with minima in Mg/Ca ratios, although these lines are not always visible”. The sentence is very tricky for a good understanding. How do you know that minima Mg/Ca ratios systematically match with these lines if they are not always visible? Please, you should explain this better. Why should I suppose then that it is true if you cannot see all lines? That is crucial.

We agree that the chronology is a crucial step. In the majority of cases we are able to identify the growth increments. In the cases we did not success, we tried every available technique (mutvei solution, fluorescence microscopy, confocal raman spectroscopy) without success. In the cases where we did not observe growth lines in the thin sections, we did still observe the on the outer shell surface.

- L. 226: I guess that these results about correlations between trace-element were extracted from figure 3. So, they should reference it in the text.

That’s correct. However, we think this figure does not add valuable information and deleted it.

**Reply to Reviewer 5**

 This manuscript’s dataset has potential to make an interesting contribution to investigations of mollusc shell elemental ratios as proxies for reconstruction of seawater parameters (i.e. temperature and salinity).
The key strength of the study is the ability to make comparisons between mollusc shell chemical proxy records and instrumental records of seawater temperature and salinity, as well as current velocity. However, these important oceanographic data are not presented within the manuscript and should be included as separate panels on a new figure. Figure 5 also should be expanded to include time-series analysis of the instrumental seawater temperature and salinity datasets (to complement the current velocity assessment), even if no clear periodicity is event. Without sight of the oceanographic data it is not possible to discern if the shell Mg/Ca records are even tracking seawater temperature. The manuscript also should be explicit with regards to the distance between the lander oceanographic instrumentation and the sampled mollusc specimens, and it is rather unclear why ARGO data are preferred to the available lander instrumentation. A site map, to support text lines 95 to 98, also would be useful.

We added the lander recorded data for temperature and salinity of the two reefs as well as spectral analysis data and a Map of the sampling locations.

We did not prefer the ARGO data. Correlations between E/Ca data and environmental data was calculated using the Lander data. ARGO data is only used because it offers a longer temperature record which we used to asses if our chronology based on growth increments fits this record.
The key weakness with the manuscript is the approach taken to convert distance along shell growth axes to time, i.e. the clearly demonstrated very weak relationship between shell Mg/Ca and seawater temperature really does not support use of Mg/Ca minima as tie points for age model development, and how interpolation between such points has been undertaken is rather unclear (i.e. constant or variable growth rates). Furthermore, it really does seem to be quite a circular argument to develop an age model using one elemental ratio (i.e. Mg/Ca) and then test for a relationship between that same proxy and seawater temperature. Also, looking at Figure 2 the ‘peak’ in Mg/Ca seems to occur in different positions relative to the ‘assumed yearly growth lines’, seemingly further negating the age model approach that has been adopted. (The yellow dashed line on Figure 2 also is not clear.) Finally, the cross section of the shell shown in Figure 4 apparently shows a clear discontinuity in shell growth, presumably due to a significant external forcing rather than a response to a slower change to external seawater conditions; could such an abrupt forcing have been changing current speed (data are not plotted to assess) or even predation? Cross sections of the other investigated mollusc specimens, even if only included within a supplementary information section, would be useful for comparative purposes and enable a more complete assessment of shell growth variability between the studied specimens.

The reviewer is right about the discontinuity in Fig 4. In this shell section a small rock was embedded in the shell. The ventral margin of these bivalves is coarse which makes it easy for small particles to get stuck. We guess one of this particles was simply overgrown by shell material. This feature almost certainly represents some kind of strong external forcing.

The age model was not developed on the basis of the Mg/Ca measurements only if the growth increments were cryptic we relied on Mg/Ca measurements. This approach was also used on coralline red algae (Halfar et al., 2000, 2008). In general we used growth lines on the shell surface and shell thin sections.
The text on lines 316 to 333 implies that an age model can be developed using the internal shell increment growth series (as shown in the bottom right panel of Figure 2), such an age model then being entirely independent of the shell elemental ratio proxies and thereby facilitating considerably more robust assessment of any relationships between shell chemistry and seawater parameters. This section, which should be called sclerochronology (and not scleromorphology) should appear after presentation of the oceanographic data and before any discussion of the mollusc shell elemental contents. It should also be possible to include graphs that present a comparison of the shell growth increment sizes (and thus growth) between the individual specimens investigated, to discuss further the comparability between the different shells for the same time period, following presentation of a robust argument for the factor(s) controlling shell increments, e.g. tidal periodicity as is mentioned within the manuscript’s text. Of course, an alternative approach to support development of an independent age model would be via use of shell oxygen-isotope ratios which could then be compared to predicted values derived from the measured seawater temperature and salinity datasets (not many studies are in a position to adopt such an approach, which this dataset actually does facilitate); such an approach also would help resolve the question as to whether there is any seasonal cessation of shell growth in the studied locations (theoretically also identifiable by counting of growth increments between growth lines, if the latter are not formed by irregular events such as predation). Mark and recapture of mollusc shells also would have helped with robust development of age models, but such an activity presumably was not completed in this study?

We added a table with the yearly growth increments for every investigated sample (Table 7). The age model was constructed using visible growth lines on the shell surface and shell thin sections.

Unfortunately, we did not mark and recapture the specimens. We added a table with the growth increment size.
The value of Figure 3 is very unclear, because the primary interest is whether the shell elemental ratios relate to oceanographic parameters, rather than to each other. Furthermore, other studies have shown that different mollusc species can exhibit differences in elemental ratios between individuals (cultured in the same experimental aquaria), such that merging all specimens together potentially hides inter-specimen variability. In fact, the caption to Figure 3 confirms this to be the case for this dataset, because two specimens are excluded as ’outliers’! If something like Figure 3 is to be presented then there should also be separate panels for each individual specimen. What would be much more useful would be to replicate Figure 2 for each of the other seven specimens, ideally plotted against an independent growth-increment (or oxygen-isotope ratio) derived age model to test whether multiple specimens from the same location(s) faithfully record consistent elemental ratios through time. Only one specimen is shown in detail (in Figure 2) at present, and plotting of elemental ratios for all individuals against time also would facilitate testing of whether the shell chemistry better follows ambient oceanographic forcing at certain times of the year, which cannot be discerned when the data are lumped together as is the case in the current manuscript. Note: a supplementary information file can be used to include multiple figures, if they will not fit within the main article.

We agree with the reviewers comment about figure 3. We deleted the figure because we do not think it adds valuable information. We also included additional figures to the supplement material which show E/Ca data against the chronology for all investigated species.

Overall, there is likely considerably more variability and interest within the dataset than is discussed at present. For example, once independent age models are developed, Figure 7 should also plot the relationships for individual specimens and not just all specimens merged together for the two reefs. Statistical testing also could be used to test for differences between specimens, and between the two sampled reefs. Furthermore, from Table 1 it is evident that all eight specimens investigated were of a comparable size (ca. 100 mm in length) and thus all adults. Could data also be included for some smaller, i.e. juvenile, individuals, so that an assessment could be made as to whether ontogeny has any influence on elemental ratios within this species? Another more minor point, was any shell periostracum removed prior to determination of shell organic content? And, can parts of Figure 4 be presented at higher magnification?

We picked the specimens to be of roughly equal size. Enough smaller specimens were not available for comparison. However, we also measured E/Ca ratios over the complete life of some specimens which did not show any ontogenetic influence.

We did not specifically clean the periostracum prior to the organic content determination. We only cleaned the shell in an ultrasonic bath for ca. 5 minutes.
Finally, the current Discussion section is not that coherent; there are long paragraphs that contain multiple short statements that are not very well interlinked. Some statements also seem rather too speculative, such as in relation to selective dissolution of calcium from a mollusc shell? Ultimately, the relationships between elemental ratios and oceanographic parameters (i.e. temperature and salinity) are very weak and thus of limited utility. The manuscript tends to overstate what might be possible, e.g. arguing that Mg/Sr ratios are better than Mg/Ca, but the r-squared values (for all data combined) only improve from 0.07 to 0.15 (from Figure 7)! There could well be more significant relationships embedded within this dataset, but such is not evident from the approach that was taken to merge all data values together.

We revised large parts of the manuscript to increase readability. Sections that we deemed overly speculative were removed. We also deleted parts that “overstate what might be possible”. This includes sections about Mg/Sr as well as the mentioned section about selective dissolution.

**References:**

Bailey, J. B.: Shell orientation terminology among the Bivalvia, Society, 83(3), 493–495, 2009.

Bergström, H.: On the central limit theorem in the case of not equally distributed random variables, Scand. Actuar. J., 1949(1), 37–62, doi:10.1080/03461238.1949.10419757, 1949.

Brooke, S. and Järnegren, J.: Reproductive periodicity of the scleractinian coral *Lophelia pertusa* from the Trondheim Fjord, Norway, Mar. Biol., 160(1), 139–153, doi:10.1007/s00227-012-2071-x, 2013.

Halfar, J., Zack, T., Kronz, A. and Zachos, J. C.: Growth and high-resolution paleoenvironmental signals of rhodoliths (coralline red algae): A new biogenic archive, J. Geophys. Res. Ocean., 105(C9), 22107–22116, doi:10.1029/1999JC000128, 2000.

Halfar, J., Steneck, R. S., Joachimski, M., Kronz, A. and Wanamaker, A. D.: Coralline red algae as high-resolution climate recorders, Geology, 36(6), 463–466, doi:10.1130/G24635A.1, 2008.

Jacobson, P.: Physical Oceanography of the Trondheimsfjord, Geophys. Astrophys. Fluid Dyn., 26(1–2), 3–26, doi:10.1080/03091928308221761, 1983.

Klein, R. T., Lohmann, K. C. and Thayer, C. W.: Sr/Ca and 13C/12C ratios in skeletal calcite of Mytilus trossulus: Covariation with metabolic rate, salinity, and carbon isotopic composition of seawater, Geochim. Cosmochim. Acta, 60(21), 4207–4221, doi:10.1016/S0016-7037(96)00232-3, 1996.

Lazareth, C. E., Le Cornec, F., Candaudap, F. and Freydier, R.: Trace element heterogeneity along isochronous growth layers in bivalve shell: Consequences for environmental reconstruction, Palaeogeogr. Palaeoclimatol. Palaeoecol., 373, 39–49, doi:10.1016/j.palaeo.2011.04.024, 2013.

López Correa, M., Freiwald, A., Hall-Spencer, J. and Taviani, M.: Distribution and habitats of *Acesta excavata* (Bivalvia: Limidae) with new data on its shell ultrastructure, Cold-Water Corals Ecosyst., 173–205, doi:10.1007/3-540-27673-4_9, 2005.

Marali, S., Schöne, B. R., Mertz-Kraus, R., Griffin, S. M., Wanamaker, A. D., Matras, U. and Butler, P. G.: Ba/Ca ratios in shells of Arctica islandica—Potential environmental proxy and crossdating tool, Palaeogeogr. Palaeoclimatol. Palaeoecol., 465, 347–361, doi:10.1016/j.palaeo.2015.12.018, 2017a.

Marali, S., Schöne, B. R., Mertz-Kraus, R., Griffin, S. M., Wanamaker, A. D., Butler, P. G., Holland, H. A. and Jochum, K. P.: Reproducibility of trace element time-series (Na/Ca, Mg/Ca, Mn/Ca, Sr/Ca, and Ba/Ca) within and between specimens of the bivalve *Arctica islandica* – A LA-ICP-MS line scan study, Palaeogeogr. Palaeoclimatol. Palaeoecol., 484, 109–128, doi:10.1016/j.palaeo.2016.11.024, 2017b.

Mucci, A. and Morse, J.: Chemistry of low-temperature abiotic calcites: Experimental studies on coprecipitation, stability, and fractionation, Rev. Aquat. Sci, 3(2–3), 217–254, 1990.

Pérez-Huerta, A. and Andrus, C. F. T.: Vital effects in the context of biomineralization, Semin. la Soc. Española Mineral., 7(1), 35–45, 2010.

Sætre, R. and Ljøen, R.: The Norwegian Coastal Current, in Proceedings of the first international conference on port and ocean engineering under Arctic conditions, Vol. II., 1971.

Sakshaug, E. and Myklestad, S.: Studies on the phytoplankton ecology of the trondheimsfjord. III. Dynamics of phytoplankton blooms in relation to environmental factors, bioassay experiments and parameters for the physiological state of the populations, J. Exp. Mar. Bio. Ecol., 11(2), 157–188, doi:10.1016/0022-0981(73)90053-1, 1973.

Schöne, B. R., Zhang, Z., Jacob, D., Gillikin, D. P., Tütken, T., Garbe-Schönberg, D., McConnaughey, T. and Soldati, A.: Effect of organic matrices on the determination of the trace element chemistry (Mg, Sr, Mg/Ca, Sr/Ca) of aragonitic bivalve shells (*Arctica islandica*) - Comparison of ICP-OES and LA-ICP-MS data, Geochem. J., 44(1), 23–37, doi:10.2343/geochemj.1.0045, 2010.

Schöne, B. R., Zhang, Z., Radermacher, P., Thébault, J., Jacob, D. E., Nunn, E. V. and Maurer, A. F.: Sr/Ca and Mg/Ca ratios of ontogenetically old, long-lived bivalve shells (*Arctica islandica*) and their function as paleotemperature proxies, Palaeogeogr. Palaeoclimatol. Palaeoecol., 302(1), 52–64, doi:10.1016/j.palaeo.2010.03.016, 2011.

Stoll, H. M., Rosenthal, Y. and Falkowski, P.: Climate proxies from Sr/Ca of coccolith calcite: Calibrations from continuous culture of Emiliania huxleyi, Geochim. Cosmochim. Acta, 66(6), 927–936, doi:10.1016/S0016-7037(01)00836-5, 2002.

Vermeij, G. J.: Molluscan marginalia: Hidden morphological diversity at the bivalve shell edge, J. Molluscan Stud., 79(4), 283–295, doi:10.1093/mollus/eyt036, 2013.
