# Supplementary material for: Compositional variability of Mg/Ca, Sr/Ca, and Na/Ca in the deep-sea bivalve Acesta excavata (Fabricius, 1779)
Source: PLoS One. 2021 Apr 30;16(4):e0245605. doi: 10.1371/journal.pone.0245605 (PMC8087087; doi:10.1371/journal.pone.0245605)
Supplement: Attachment — Submitted filename: Reply_to_reviewer_002.docx [file pone.0245605.s011.docx]

Reviewer #2: Overall the paper is improved but still needs work to be publishable. The statistical aspects of the paper need to be addressed better but should not change the overall results.

General observations:
I suspect growth-related effects are a source of the problem with these bivalves, have the authors separated out faster and slower growing shells and compare their chemistry that way? The growth-related effect on biological carbonate material have been explored in corals, first documented by McConnaughey, T. (1989) for δ18O and deVilliers et al. (1994) for Sr/Ca. See Kuffner et al. (2017) figure 3 for an example of how to make this assessment.
We thank the reviewer for that valuable comment.
We did pick the investigated shells to be of roughly equal size to ensure they are in a comparable life cycle. We did the assessment you suggested (based on Kuffner et al. 2017) but we do not observe a regular trend of the residuals as in your suggested paper. However, since we only report annual extension rates and our temperature regression only spans one year we cannot exactly recreate the method by Kuffner et al. 2017
Rev. 2 : The study sample the outer increments or younger part of the shell that is slower growing thus, slower growth could be driving El/Ca. Why didn’t the authors sample the older faster growing portions of the shell? I understand in tree-ring studies that the early years of growth have a growth trend that is removed for tree-ring increment studies. However, shells are not trees and this study is looking at chemistry not growth increments. I would think you would sample the initial growth years when growth is faster to avoid the slower growth- related effects. This should eb explained in the introduction and study design and then discussed in the discussion section.
The main reason we are sampling the younger shell parts is because we only have environmental data for 07/2013 – 07/2014. For samples that were picked alive in 07/2014 we can be sure that the last growth increments relates to the recorded environmental data.
In addition, inorganic precipitation experiments show that, for Mg and Sr, the calcification process happens in equilibrium with the calcification fluid at low calcification rates [1]. Therefore, its preferable to use slow growing shell areas instead of fast growing areas.
Rev. 2:Next, is replication, I assume the shells are all collected at the same time so the authors should be able to assess reproducibility of the El/Ca signals among the shells from each location and then if there is any correlation between locations. If the shells have a same signal, then the forcing is environmental, if not biological. This is not explored in the statistical tests of the study.
All shells were collected in summer 2014. We had this data in a previous version but deleted upon suggestion of another reviewer. We added another figure to the supplementary material that shows the intra-reef variability of the E/Ca ratios.
Rev. 2:Correlation must be reported with the n used for the significance testing. Correlations < 0.1 are dubious regardless of the p-valve because “n” is degrees of freedom in the correlation equation, not number of observations. Laser Ablation sampling generates a lot of data; thus, I suspect the n values are high (>1000) and thus why the authors’ p values are low suggesting significance. The authors data has serial correlation in it, and this MUST be considered in the evaluation of correlation significance determine with degrees of freedom, NOT the number of observations. Looking at Figure 5 there is 6 years of data, so the authors’ degrees of freedom would be close to 12. However, if the authors determine the degrees of freedoms, the authors will find these correlation results are probably different and possibly not significant. The authors are can use the Runs test or best method is Ebisuzaki, W. (1997). "A Method to Estimate the Statistical Significance of a Correlation When the Data Are Serially Correlated." Journal of Climate 10(9): 2147-2153. If the correlations are weak and the authors are not going to show the scatterplots in the main paper, then why mention the correlation and their details in the abstract? Same for the T-test and f-test (in Table 3), the n should be degrees of freedom. With more than 1000 data points almost any r will be significant (r >0.062 is significant at 5% level). See Zwiers, F. W. and H. von Storch (1995). "Taking serial correlation into account in tests of the mean." Journal of Climate 8: 336-351.
You are right that the total data for each sample is serially correlated. However, the data we used to calculate the correlation is not because it only includes one annual cycle (see S5). We added degrees of freedom to the tables (L268, L277,L307, L313)
Other statistical test questions that are not clear:
Rev. 2: Table 2, 3, 4 How many annual cycles or years were compared for these shells, i.e. are the authors comparing the same time intervals or varying time intervals for all the shells. Looking a Fig S7, the data spans different number of years in the shells. The same interval and same amount of data of time should be made for the comparison tests (correlation, t-test, f-test, etc.).
In Table 2 and 3 we used all available data. In Table 4 we used only the last annual cycle. We redid the analysis with the same time interval for all shells and found no differences in the results.
Rev. 2:Line 270 Table 4 reports means, and the T-test is for means only. Revise “Significant mean differences are observable for every E/Ca in all measured samples.”
Corrected L280
Rev. 2: Explain how the multiple linear regression was performed, give citation or software used.
We added a section about the statistical analysis, which program we used and the used tests. L190
“All statistical calculations were conducted with OriginPro 2020. We used the T-test to compare means of different populations and a linear regression model to investigate the relationship between predictor and response variables….”
Rev. 2:Add a table that gives the time interval each shell spans. Explain what interval for each shell was used for statistical tests. For a robust statistical assessment, the same time interval should be assessed for each shell not 5 years in one and 2 years in another. Looking at Table 7 the time interval for each shell varies and the common interval is just two years when excluding 6R. The authors could expand the windows and see how statistical tests vary with more years but less shells.
The time interval of each sampled shell is given in table 7. For the correlation between E/Ca and Salinity-temperature we used the same time interval for each shell (1 year).
Rev. 2:The statistical results and text in the abstract are distracting (i.e., r=0.05, p<0.01), just tell the reader what you found.
We added the statistical results upon suggestion of another reviewer. However, we agree that it is distracting.
Rev. 2: was the cleaning test and organic matter tests for whole shell or a similar interval as the LA-ICP-MS data? See previous comments about time intervals in shells and statistical tests.
We used the same interval for the cleaning test as for the LA-ICP-MS data.
Rev. 2:Authors should carefully proof reader for typos, spelling mistakes, punctuation, and figure and tables numbers are correct.
We carefully eliminated occurring typos and wrong table – figure numeration.
Rev. 2:The authors have made their data available in the publication but they should also make it available in the community accepted repositories at NOAA Paleoclimatology (Khider et al. 2019) https://www.ncdc.noaa.gov/data-access/paleoclimatology-data or Pangaea https://www.pangaea.de/.
We are currently preparing the data to be uploaded to Pangaea
Rev. 2:Line 271 Same as previous comment, “Mean Mg/Ca are lower in the microgranular shell layer.” Fix elsewhere. Looking at Fig. 5 the Mg/Ca are not always lower in the microgranular shell layer.
Corrected to mean Mg/Ca L281
Rev. 2:Line 269-278 The authors should also reference and discuss the plots of the data in the Fig. 5 and Fig S7.
We added references to these figures.
Specific items to address:
Rev. 2: Line 17 revise for plural agreement “parameters”.
Corrected. L17
Rev. 2: Abstract and elsewhere. Correlations must be reported with the degrees of freedom used to determine p-values. Where these for the Fibrous or Microgranular parts of the shell? Revise abstract to be more informative to the reader. It is OK to report negative results, these are important so others do not repeat your negative findings.
We revised the abstract according to your suggestion and added DF to reported correlations in the tables.
Rev. 2:Line 63 Revise for missing verb “…first phase lasts until the bivalve has built 18-22 increments…” Does an increment – one year? If so, explain this.
Yes, 18 increments equal 18 years [2]. We made the statement clearer. L66
Rev. 2:Line 64 “crammed” means to be “completely fill” is a not a good word to use here, use a more scientifically descriptive word like “tightly aligned”. Delete “much” it is not needed.
Corrected. L68
Rev. 2:Line 78-79 This is not a complete sentence, revise or use correct punctuation.
Corrected.
“Whether this assumption holds true for other species has to be further investigated.L81
Rev. 2:Line 83 The elemental ratios are written as a ratio Mg/Ca, do not say “ratio” afterwards, it is redundant. The “/” means divides by or ratio. Fix throughout the paper.
You are right that adding the word “ratio” might be redundant, however this redundancy is often used in PLOS ONE articles.

“Whether using the δ^18^O signatures or Mg/Ca ratios from foraminiferal tests…” [3]

“ The molar magnesium/calcium (Mg/Ca) ratio (mMg/Ca) of seawater has varied between 0.5 and 5.3 through the current Phanerozoic eon [[1]](https://journals.plos.org/plosone/article?id=10.1371/journal.pone.0091021#pone.0091021-Ries1).”[4].

We adjusted the wording in some instances but we think that it increases the readability and makes some statements better understandable.
Rev. 2:Line 114 “macroscopically visible” is redundant macroscopic means visible without magnification and it is an adjective, macroscopically is not a word.
We want to object that the term „macroscopically visible“ is commonly used in PLOS ONE articles in the same sense we used it, being that the features are visible with the bare eye..

„The macroscopically visible appearance of the satellite cluster is indicated with a red circle.“[5]

“The extent of macroscopically visible cell density observed across” [5]

“…, no macroscopically visible alterations of the skin were found 2.5 weeks after infection ([Fig. 1, A1 and A2](https://journals.plos.org/plosntds/article?id=10.1371/journal.pntd.0002968#pntd-0002968-g001)).”[6]
Rev. 2:Line 133 “The data shown here” Where are these data the authors say are shown? I think the authors mean “the data used”.
Corrected. L136
Rev. 2: Methods section grammar needs to be addressed. The first section “Sampling” is written in past tense and the next section “Oceanographic data” starts in present tense and then switches to past tense. Use one tense (past) throughout the methods section.
We adjusted the wording to have a regular tense.
Rev. 2: Line 155 Fig. 1 is the study site map, the authors mean Fig. 2B.
Corrected. L158
Rev. 2: Line 192 Need space between fig. and number. Fix elsewhere (line 227).
Corrected
Rev. 2: Line 214 in correct use of “/” in “aragonite/calcite” replace with a hyphen.
Corrected. L223
Rev. 2: The captions and figure # seem to not correspond for Fig 4 and 5. The caption for fig. 5 seems to be to be for the figure labeled fig 5 and vice versa. Line 215 refers to fig 4 but the caption for fig 4 does not make sense, it looks to be referring to the fig labeled fig. 5.
Corrected
Rev. 2: Line 215 What do the authors mean by “visible sculpture”? the jagged edge? A “sculpture” is a work of art of in two- or three-dimensional representative or abstract forms. I do not think this is the right word.
Corrected to jagged edge.L224
Rev. 2: Line 252 What exactly do the authors mean by “There is no resolvable difference in Sr/Ca ratios between the two sample sites”? Did the authors do a T-test for means and F-test for variances? Do they look like they plot of top of each other the same? If so, show this. Do they co-vary , do the growth intervals appear to be the same. Explain this better with no detail.
We want to express here that based on a T-test there is no difference in the mean Sr/Ca ratio between the two reefs. However, we deleted the statement because it adds no necessary information.
Rev. 2: Line 282 Revise “…own observations, we assume the growth lines were produced on an annual basis.”
Corrected. L292
Rev. 2: Line 289 Revise “…temperature, as well as Mg/Ca,…” Spelling mistake
Corrected
Rev. 2: Line 303 period is missing at end of sentence.
Corrected
Rev. 2: Line 358-363 The authors should be cautious in their interpretation. They tested mean values between the shell layers. Looking at Fig. 5 the two layers appear to track each other in all three e/Ca. A correlation between the layers how help make this assessment.
We are not entirely sure what the reviewer is suggesting here. Indeed, the two layers track each other in every measured E/Ca ratio but the mean ratios are different. These differences are most likely caused by the different crystal morphology in the layers.
Rev. 2: Figure 4 caption and Figure 2 Explain and show what the authors mean by the “commissure line on the shell”.
In Figure 4 we mean the ventral margin of the shell. We changed the wording to be clearer. We cannot find the term in Figure 2.
Rev. 2: Table 1 there is “229” in the bottom right cell, not sure that is supposed to be there.
The number is not visible in the .docx file. It seems to be added during the PDF conversion
Rev. 2: Supplemental Figure 7 is too small to read the values and look at the shells, please put these on multiple pages and enlarge images figure Fig. 5. Same for line 254. Make the scale for El/Ca for each shell the same so the reader can compare visually. These figures should show microgranular and fibrous data.
We updated the figure according to your suggestions.
Rev. 2: Table 4 How many annual cycles or years were compared for these shells, i.e. are the authors comparing the same time intervals or varying time intervals for all the shells. Looking a Fig S7, the data spans different number of years in the shells. The same interval and same amount of data of time should be made for the comparison tests.
The data compared here consists of different time interval for each sample. However, we only compare the data between the microgranular and fibrous shell section which spans the same interval for each sample.
Rev. 2:Table 5 Explain in the caption. Explain in the caption how All samples was determined, the same time intervals, or just all the data produced? If different intervals, then sample size needs to be considered.
For the correlation with temperature and salinity we used the same time interval. The values given under all samples relate to a combined regression with all samples in the same time interval.
Rev. 2:Table 6 Explain in results or in the methods how the multiple linear regression was performed, give citation or software used.
We added a section about the statistical analysis, which program we used and the used tests.
Rev.2: Line 389 Spelling of “therefor” is incorrect.
Corrected. L397
Rev. 2:Line 398 What is the spatial-temporal resolution of the authors’ study? Add this to the table with the time interval present in the data for each shell that I requested.
For the statistical analysis we interpolated the data to a daily resolution.
Rev. 2:excavata are not well suited for paleo temperature and salinity reconstructions due to weak correlations between proxy trace elements and these environmental variables,…” Same for Line 547
Revised. “…section indicate that elemental ratios in *A. excavata* are not well suited for paleo reconstructions due to weak correlations between proxy trace elements and the environmental variables temperature and salinity…”
Rev. 2:Conclusions- I like the discussion and conclusions- use these to revise the abstract.
We revised the abstract.

Reviewer #3: The authors addressed my concerns from the previous revisions. The data and the study will be useful for other researchers and now that the others have more carefully assessed the physiological/vital effects that obscure the recorded environmental signals in the shell elemental ratios.

We want to thank the reviewer for the comprehensive comments which drastically improved this manuscript.

Reviewer #4: I grateful to editor the opportunity to review this manuscript. After reading it, unfortunately I should recommend its rejection. They have done a very good job and the methodology seems very strong. However, I am not very confident in the value of this manuscript for a very high-quality journal like Plos One.

The study conducted by the authors seems based on a very strong-methodology, but the results obtained have not implications for future climate studies. Please, take into account that it is not my subjective opinion, authors are who conclude the manuscript writing: "it can be stated that Mg/Ca, Sr/Ca and Na/Ca ratios in A. excavata are unlikely to be good proxies for environmental reconstructions at this point". They also admit it in the discussion section: “…these results indicate that Mg/Ca, Sr/Ca and Na/Ca are unlikely to be used for environmental reconstruction”. I agree, these E/Ca ratios are not reliable climate proxies. In four shells, they found a correlation lower than 0.1 between SST and Mg/Ca ratios. Mean correlations between E/Ca ratios and SST or Salinity are 0.06, 0.02, 0.17, 0.02, 0.04 and 0.12. I am so sorry, but these coefficients are not reporting a weak correlation as authors said, they are actually showing an absent of correlation. In this same sense, the authors are who admit that only 6% of the variability of Mg/Ca ratios can be explained by SST changes through mollusk life span. It is not possible to say at same time that 0.06 is a weak correlation while then they say that magnesium precipitation is only driven by SST in a 6%. In summary, manuscript have valuable data, a strong methodology, but the results, conclusions and specially its implications for future studies are not good enough for a very high impact journal like this.

In any case, if editor decide to admit it or if the other reviewers have favorable opinions, please check the manuscript again very carefully, there are several mistakes. Some examples: L. 155: Fig. 1 should be Fig. 2. L. 287. You said that maximum correlation value is 0.64, but in the table 5 the highest value is 0.63. L. 389. Please, correctly write “therefore”.

We adjusted the wording to be more careful with the reported results and corrected your mentioned errors

References

1.	Gabitov RI, Sadekov A, Leinweber A. Crystal growth rate effect on Mg/Ca and Sr/Ca partitioning between calcite and fluid: An in situ approach. Chem Geol. 2014;367: 70–82. doi:10.1016/j.chemgeo.2013.12.019

2.	López Correa M, Freiwald A, Hall-Spencer J, Taviani M. Distribution and habitats of *Acesta excavata* (Bivalvia: Limidae) with new data on its shell ultrastructure. Cold-Water Corals Ecosyst. 2005; 173–205. doi:10.1007/3-540-27673-4_9

3.	Stainbank S, Kroon D, Rüggeberg A, Raddatz J, De Leau ES, Zhang M, et al. Controls on planktonic foraminifera apparent calcification depths for the northern equatorial Indian Ocean. PLoS One. 2019;14: e0222299. doi:10.1371/journal.pone.0222299

4.	Higuchi T, Fujimura H, Yuyama I, Harii S, Agostini S, Oomori T. Biotic Control of Skeletal Growth by Scleractinian Corals in Aragonite–Calcite Seas. Roberts JM, editor. PLoS One. 2014;9: e91021. doi:10.1371/journal.pone.0091021

5.	Shelswell KJ, Beatty JT. Coordinated, Long-Range, Solid Substrate Movement of the Purple Photosynthetic Bacterium Rhodobacter capsulatus. Otto M, editor. PLoS One. 2011;6: e19646. doi:10.1371/journal.pone.0019646

6.	Bolz M, Ruggli N, Ruf M-T, Ricklin ME, Zimmer G, Pluschke G. Experimental Infection of the Pig with Mycobacterium ulcerans: A Novel Model for Studying the Pathogenesis of Buruli Ulcer Disease. Small PLC, editor. PLoS Negl Trop Dis. 2014;8: e2968. doi:10.1371/journal.pntd.0002968

7.	Klishko OK, Lopes-Lima M, Bogan AE, Matafonov D V., Froufe E. Morphological and molecular analyses of Anodontinae species (Bivalvia, Unionidae) of Lake Baikal and Transbaikalia. Vermeij GJ, editor. PLoS One. 2018;13: e0194944. doi:10.1371/journal.pone.0194944

8.	Yates AM, Neumann FH, Hancox PJ. The Earliest Post-Paleozoic Freshwater Bivalves Preserved in Coprolites from the Karoo Basin, South Africa. Farke AA, editor. PLoS One. 2012;7: e30228. doi:10.1371/journal.pone.0030228

9.	Purroy A, Šegvić-Bubić T, Holmes A, Bušelić I, Thébault J, Featherstone A, et al. Combined Use of Morphological and Molecular Tools to Resolve Species Mis-Identifications in the Bivalvia The Case of Glycymeris glycymeris and G. pilosa. Vermeij GJ, editor. PLoS One. 2016;11: e0162059. doi:10.1371/journal.pone.0162059
